# Redox-Active Profile Characterization of *Remirea maritima* Extracts and Its Cytotoxic Effect in Mouse Fibroblasts (L929) and Melanoma (B16F10) Cells

**DOI:** 10.3390/molecules200711699

**Published:** 2015-06-25

**Authors:** Grace Anne A. Dória, Anderson R. Santos, Leonardo S. Bittencourt, Rafael C. Bortolin, Paula P. Menezes, Bruno S. Vasconcelos, Rebeca O. Souza, Maria José V. Fonseca, Alan Diego C. Santos, Shanmugam Saravanan, Francilene A. Silva, Daniel P. Gelain, José Cláudio F. Moreira, Ana Paula N. Prata, Lucindo J. Quintans-Júnior, Adriano A. S. Araújo

**Affiliations:** 1 Departament of Pharmacy, Federal University of Sergipe, Av. Marechal Rondon, Jardim Rosa Elze, 49100-000 São Cristóvão, Sergipe, Brazil; E-Mails: gracedoria@hotmail.com (G.A.A.D.); andersonfarmacia@yahoo.com.br (A.R.S.); paulamenezes_16@yahoo.com.br (P.P.M.); bruninho_farma@hotmail.com (B.S.V.); saranflora04@gmail.com (S.S.); farmsilva@hotmail.com (F.A.S.); 2 Departament of Biochemistry, Federal University of Rio Grande do Sul, 90040-060 Porto Alegre, Rio Grande do Sul, Brazil; E-Mails: lsbittencourt@hotmail.com (L.S.B.); rafaelbortolin@hotmail.com (R.C.B.); dgelain@yahoo.com.br (D.P.G.); 00006866@ufrgs.br (J.C.F.M.); 3 Departament of Pharmacy, University of São Paulo, 14040-900 Ribeirão Preto, São Paulo, Brazil; E-Mails: rebecapharma@yahoo.com.br (R.O.S.); magika@fcfrp.usp.br (M.J.V.F.); 4 Departament of Physiology and Chemistry, Federal University of Sergipe, 49100-000 São Cristóvão, Sergipe, Brazil; E-Mails: alandiego0@yahoo.com.br (A.D.C.S.); lucindo_jr@yahoo.com.br (L.J.Q.-J.); 5 Departament of Biology, Federal University of Sergipe, 49100-000 São Cristóvão, Sergipe, Brazil; E-Mail: apprata@yahoo.com.br

**Keywords:** *Remirea maritima*, free radicals, cellular viability, oxidative stress, cytotoxicity, total phenol

## Abstract

*Remirea maritima* is a tropical plant with a reticulated root system belonging to the family Cyperaceae, also known to have biologically active secondary metabolites. However, very few data on *R. maritima’s* biological actions are available and there are no reports regarding the redox-active profile of this plant. In this study, we examined the total phenolic content of *Remirea maritima* hydroalcoholic (RMHA) extracts, redox properties against different reactive species generated *in vitro* and their cytotoxic effect against fibroblasts (L929) and melanoma (B16F10) cells. Total reactive antioxidant potential index (TRAP) and total antioxidant reactivity (TAR) results revealed that RMHA at all concentrations tested showed significant antioxidant capacity. RMHA was also effective against hydroxyl radical formation, reduction of Fe^3+^ to Fe^2+^ and in scavenging nitric oxide (NO) radicals. *In vitro*, the level of lipid peroxidation was reduced by RMHA extract and the data showed significant oxidative damage protection. The RMHA cytotoxicity was evaluated by a neutral red assay in fibroblast (L929) and melanome (B16F10) cells. The obtained results showed that the RMHA (40 and 80 µg/mL, respectively) reduced 70% of the viable cells. In conclusion, this study represents the first report regarding the antioxidant and anti-proliferative potential of *R. maritima* against B16F10 melanoma cells.

## 1. Introduction

The pathogenesis of numerous chronic diseases has been related with unbalanced levels of free radicals in systemic tissues [1]. Epidemiological surveys indicate that the diet plays an important role in preventing chronic diseases due to the antioxidants it supplies [2,3,4].

Plant-based foods contain significant amounts of phytochemicals which possess numerous health benefits [5]. These phytochemicals are mostly phenols, which can be divided into at least 10 types depending on their basic structure. The flavonoids represent the most important type of polyphenolic compound [6]. Several studies have previously reported that many of these phytochemicals have antioxidant activity, and anticarcinogenic and antimutagenic effects by inhibiting cell proliferation [7,8,9,10]. Furthermore, antioxidants are the main compounds considered to exert anti-inflammatory, anti-aging and health-promoting effects in the human body [11].

*Remirea maritima* Aubl. is a tropical plant belonging to the family Cyperaceae with a reticulated root system that develops rhizomes below the sand. This family is known to contain biologically active secondary metabolites such as xanthones, chalcones, coumarins, flavonoids, triterpenes, benzofurans, *etc.* [12]. *R. maritima* is popularly employed in medicinal preparations for the treatment of diarrhea, kidney disease, fever, pain, and inflammatory processes [13,14]. At the moment, there are few reported studies about the chemistry and pharmacology of *R. maritima* plants. Recently, the aqueous extract was evaluated for chemical composition, thiobarbituric acid reactive species (TBARS) and nitric oxide (NO) assays, anti-inflammatory and antinoceptive properties. The phytochemical profile of the aqueous extract showed three flavone glycosides (vitexin, isovitexin and luteolin) as components and it presented an effective anti-inflammatory activity and reduced NO and lipid peroxidation [15]. The phytochemical characterization, and antitumor effects of RMHA were studied by our group and we have reported vitexin, isovitexin, luteolin and cafeoil as components and it also showed antitumor activity [16]. The work heported herein now provides the first data regarding the complete *in vitro* redox-active properties and the cytotoxicity of *R. maritima* hydroalcoholic extracts. 

## 2. Results

### 2.1. Determination of Total Phenolic Content (TPC)

The total phenolic content in RMHA was analyzed by the Folin-Ciocalteau method and estimated by comparison with a standard phenolic compound (gallic acid). Total phenolic content of the extract was 0.58 mg gallic acid equivalents/g of RMHA extract.

### 2.2. Total Reactive Antioxidant Potential (TRAP) and Total Antioxidant Reactivity (TAR)

**Figure 1:**
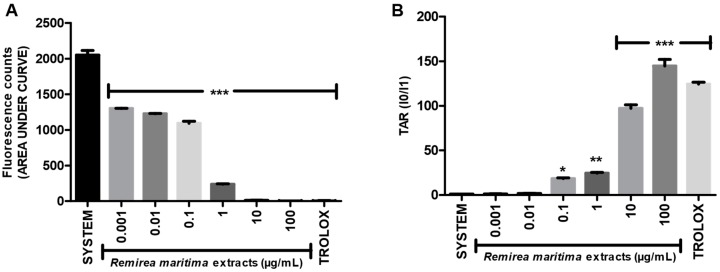
(**A**) Total reactive antioxidant potential (TRAP) assay; (**B**) Calculated total antioxidant reactivity (TAR) values. Trolox^®^ (75 µg/mL) was used as standard antioxidant. Bars represent mean ± SEM. * *p* < 0.05, ** *p* < 0.01, *** *p* < 0.001 (1-way ANOVA followed by Tukey’s multiple comparison *post hoc* test).

The general antioxidant capacity of RMHA extract was evaluated by the TRAP and TAR assays. In the TRAP assay, RMHA concentrations ranging from 0.001 to 100 µg/mL showed a significant dose-dependent antioxidant effect (Figure 1A). At the concentrations of 10 and 100 µg/mL, RMHA extract revealed the best results, similar to those of Trolox^®^ (75 µg/mL) used as a reference antioxidant. RMHA concentration ranges 0.1 to 100 µg/mL also showed a significant antioxidant capacity in the TAR assay (Figure 1B) and a concentration of 100 µg/mL RMHA extract showed better results than the control and Trolox^®^ groups.

### 2.3. Hydroxyl Radical-Scavenging Activity

**Figure 2:**
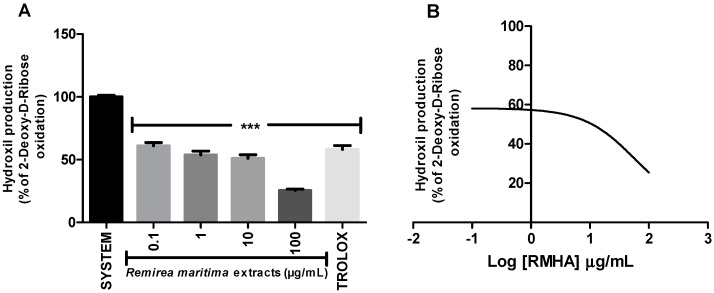
(**A**) Hydroxyl radical-scavenging activity quantified using the oxidative *in vitro* degradation of 2-deoxyribose; (**B**) Log IC_50_ of RMHA hydroxyl scavenging activity. TROLOX^®^ (75 µg/mL) was used as standard antioxidant. Bars represent mean ± SEM. *** *p* < 0.001 (1-way ANOVA followed by Tukey’s multiple comparison *post hoc* test).

Degradation of 2-deoxyribose (2-DR) by hydroxyl radicals generated *in vitro* by a Fenton reaction was used to evaluate the hydroxyl radical scavenging capacity of RMHA extract. Concentrations of 0.1–100 µg/mL of RMHA extract were effective in scavenging the hydroxyl radicals (Figure 2A), showing similar results to Trolox^®^ (75 µg/mL). RMHA extract at 100 µg/mL was better in reducing hydroxyl radicals than Trolox^®^ (75 µg/mL). We also calculated the IC_50_ of RMHA hydroxyl scavenging activity, which gave a value of 13.1 µg/mL (Figure 2B).

### 2.4. Ferric Reducing Antioxidant Power (FRAP) and Fe^2+^ Chelation Assay

**Figure 3:**
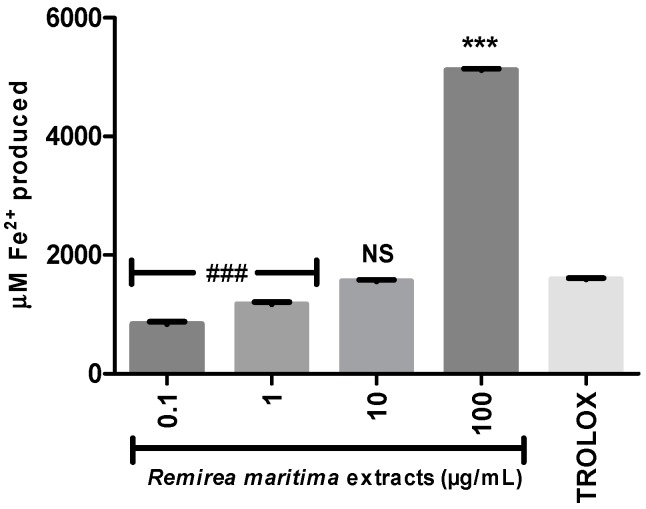
Ferric Reducing Antioxidant Power (FRAP). Trolox^®^ (75 µg/mL) was used as standard antioxidant. **###** less efficiency than Trolox^®^ (*p* < 0.0001); NS Non-significant compared to Trolox^®^; *** higher efficiency than Trolox^®^ (*p <* 0.0001) (1-way ANOVA followed by Tukey’s multiple comparison *post hoc* test); bars represent mean ± SEM.

To evaluate whether RMHA was able to reduce Fe^3+^ to Fe^2+^, we performed the FRAP assay. All tested concentrations are able to reduce iron, however at a concentration of 10 µg/mL RMHA showed similar results to Trolox^®^ (75 µg/mL) and at a concentration of 100 µg/mL RMHA was more efficient than the standard antioxidant in reducing the iron (Figure 3).

**Figure 4:**
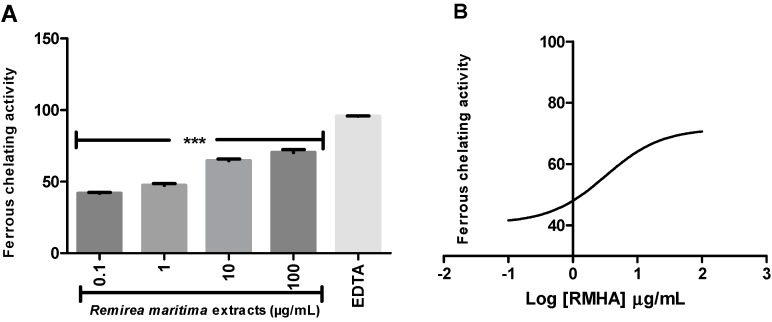
(**A**) The ability of RMHA extracts in prevent the Fe^2+^-ferrozine complex formation, thus decreasing the red color compared to control; (**B**) Log IC_50_ graph. EDTA was used as standard chelating agent. Bars represent average ± SEM of three independent experiments. *** *p* < 0.001 (1-way ANOVA followed by Tukey’s *post-hoc* test).

Ferrous ion-chelating activity is an important indicator of oxidative stress involving Fe^2+^ ion. Ferrozine forms red complexes with Fe^2+^ quantifiable by spectrophotometry. In the presence of chelating agents the complex formation is disrupted or prevented, resulting in a decrease in the red color of the complex. Measurement of the color reduction allows estimation of the metal chelating activity of the analysed extract. All RMHA extracts were efficient in chelating ferrous ion (Figure 4A), thus preventing the aforementioned Fenton reaction and at low IC_50_ (2.4 µg/mL) (Figure 4B).

### 2.5. Nitric Oxide (NO**^·^**) Scavenging Activity

The ability of RMHA to scavenge NO was measured by quantifying the production of nitrite derived from sodium nitroprusside (SNP) by the Griess reaction.

**Figure 5:**
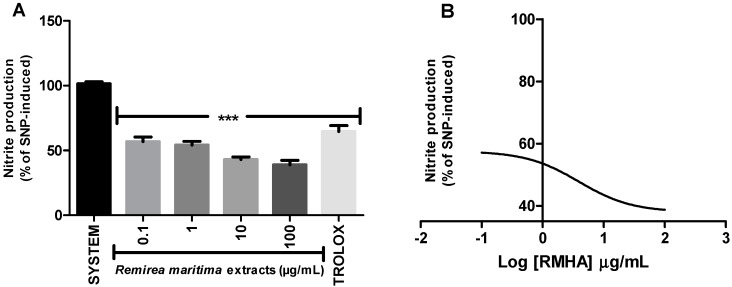
Nitric oxide (NO) scavenging assay (**A**) NO was generated from spontaneous decomposition of sodium nitroprusside (SNP) in the presence O_2_, producing NO_2_^‒^ ions which were measured by the Griess reaction. Trolox^®^ (75 µg/mL) was used as standard antioxidant; (**B**) Log IC_50_ of RMHA NO scavenging activity. Bars represent mean ± SEM. *** *p* < 0.001 (1-way ANOVA followed by Tukey’s multiple comparison *post hoc* test).

All tested concentrations of RMHA (0.1–100 µg/mL) showed NO radical scavenging capacity (Figure 5A). RMHA was more active than Trolox^®^. The IC_50_ value of the RMHA scavenging effect upon SNP-induced NO production was also calculated, giving a result of 4.04 µg/mL (Figure 5B). 

### 2.6. Thiobarbituric Acid Reactive Species (TBARS)

**Figure 6:**
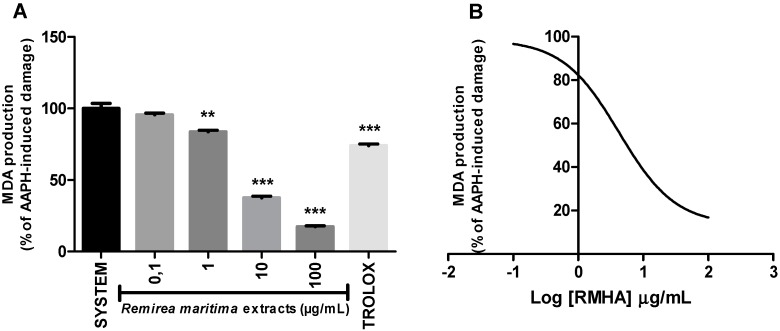
TBARS assay *in vitro*. (**A**) Effect of different concentrations of RMHA on lipid peroxidation; (**B**) Log IC_50_ of RMHA lipid peroxidation activity. Trolox^®^ (75 µg/mL) was used as standard antioxidant. Bars represent mean ± SEM. ** *p* < 0.01, *** *p* < 0.001 (1-way ANOVA followed by Tukey’s multiple comparison *post hoc* test).

The capacity of RMHA to protect from oxidative damage by lipid peroxidation was measured by quantification of thiobarbituric acid-reactive substances (TBARS) generated by AAPH in a lipid-rich incubation medium. The effect of different concentrations of RMHA on lipid peroxidation is shown in Figure 6A. At concentrations of 1–100 µg/mL, RMHA reduced the AAPH-induced lipid peroxidation when compared with standards. At concentrations of 10–100 µg/mL, RMHA shows better protection against lipid peroxidation than Trolox^®^. Figure 6B shows the IC_50_ value describing the ability of RMHA to prevent lipid peroxidation, which was 4.04 µg/mL.

### 2.7. Cytotoxicity Assay

**Figure 7:**
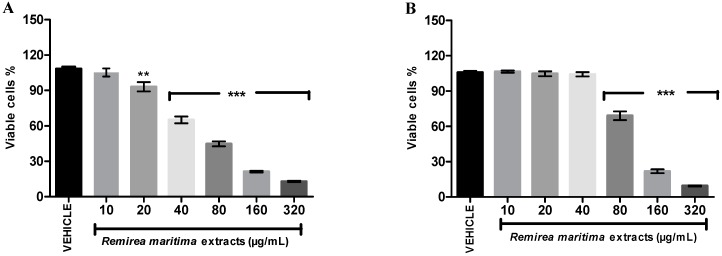
Effect of RMHA on cell viability at different concentrations assessed by a neutral red assay. (**A**) L929—fibroblast cells and (**B**) B16F10—melanoma cells. Control group received distilled water (1:1). *n* = 4, ** *p* < 0.01, *** *p* < 0.001 (1-way ANOVA followed by Tukey’s multiple comparison *post hoc* test).

Cytotoxicity evaluation was conducted in two strains of cells, normal L929 fibroblast cells (Figure 7A) and melanoma B16F10 tumor cells (Figure 7B).

RMHA showed cytotoxic activity with an IC_50_ value of 70.96 µg/mL (65.59–77.96) in L929 cells and an IC_50_ value of 108.30 µg/mL (101.90–115.00) in B16F10 cells. At 80 µg/mL RMHA exhibited a significant reduction of viable melanoma cells, while a significant decrease of normal cells only started to appear at a 20 µg/mL concentration of RMHA.

## 3. Discussion

Numerous reports suggest that reactive oxygen species (ROS) and reactive nitrogen species (RNS) are involved in the pathogenesis of several diseases such as cancer, inflammatory diseases, neurodegenerative disorders, hypertension, diabetes and cardiovascular disorders [17,18,19,20,21].

ROS/RNS are free radicals, which are atoms or groups with highly reactive unpaired electrons [22]. ROS represent a class of the most important free radicals produced in living systems. Oxygen is essential for the survival of aerobic beings, however, the process of reduction of oxygen and water in generating ATP can produce ROS such as superoxide (·O_2_**^−^**) as intermediates and non-free radicals, such as hydrogen peroxide (H_2_O_2_), which causes potential damage to cells [19].

On the other hand, ROS/RNS, under normal physiological conditions, participate as regulatory molecules, intracellular messengers, tissue protection against further insults, controlling cell proliferation by inducing apoptosis and defending against cancer [23,24,25]. Thus, ROS, as byproducts of oxygen metabolism, are constantly produced in the human body and removed by antioxidant defenses by different pathways, antioxidant compounds, antioxidant enzymes, proteins and exogenous components as carotenoids, flavonoids, and other phenolic compounds, including vitamin E, vitamin C and coumarin derivatives [26,27,28]. Briefly, oxidative stress can play an important role in the pathogenesis of diseases and that can be counteracted through the natural scavenging system which balances ROS generation and elimination [29].

Antioxidants are compounds that at low concentrations prevent or interfere in the oxidation of oxidisable substrates such as DNA, lipids, and proteins. In biological systems, there are two main classes of the antioxidants systems: the enzymatic and nonenzymatic [30] antioxidant systems.

Several studies have shown that the redox activity from natural antioxidants is related to the total content of phenolic compounds [31,32,33]. Generally these compounds are present at high levels in vegetables and fruits, which thus have the capacity to reduce oxidative stress [6,34,35].

The TPC of RMHA was evaluated using the Folin–Ciocalteu reagent. The plant extract showed a higher level of phenolic compounds (582.5 mg gallic acid/g RMHA extract) compared to the TPC of several other well known plants, such as *Acacia podallyriifolia* (206.4–338.5 mg gallic acid/g) [36], *Terminalia brasiliensis*, *Terminalia fagifolia*, *Copernicia cerifera*, *Cenostigma macrophyllum*, *Qualea grandiflora* (250.0–763.6 mg gallic acid/g) [37], *Limnocharis flava*, *Momordica charanti*, *Eugenia siamensis*, *Piper betel*, *Acacia catechu* (5.4–180.5 mg gallic acid/g) [38] and *Morinda citrifolia* (196.8 mg gallic acid/g) [39]. This high TPC is what led us to investigate the redox-active profile of RMHA extracts since phenolic compounds are described to have antioxidant properties [40,41]. The redox properties of RMHA were evaluated using different approaches to understand the possible effects in different reactive species-generating sources, such as superoxide radical (O_2_^.^), hydroxyl (**^.^**OH), peroxyl, nitric oxide (**^.^**NO) and redox active metals. 

TRAP/TAR assays showed the general antioxidant ability of RMHA. TRAP and TAR are different methods employed to estimate the general antioxidant potential and capacity of samples *in vitro*. In Figure 1A, the bars represent the area under the curve of a kinetic measurement of AAPH-induced luminescence during 120 min (TRAP measurement); in the TAR assay, the immediate effect of the addition of an antioxidant compound in the free radical-induced chemiluminescence is measured [42]*.* The RMHA extracts was able to maintain the AAPH-induced free radical production inhibited during 120 min (TRAP assay) at all tested concentrations (Figure 1A). Regarding TAR measurements, RMHA showed high efficiency in inhibiting the AAPH-induced free radical production when immediately added to a system at concentrations ranging from 0.1 to 100 µg/mL (Figure 1B). As we can see in Figure 1A, the concentrations of 0.001 and 0.01 µg/mL presented suitable antioxidant kinetic behavior, but low antioxidant quality when the TAR measurements were analyzed (Figure 1B), thus justifying why such concentrations were chosen for the other analyses described in this study. 

Peroxyl radicals formed in the TRAP reaction participate in the initiation and propagation steps of lipid peroxidation and can produce cytotoxic and genotoxic products. These toxic products promote cell damage involved in the pathogenesis of many diseases [1,43].

Moreover, RMHA was capable of quenching hydroxyl radicals. The extract was thus highly effective in inhibiting 2-DR hydroxyl-induced damage. Doses of 0.1 to 100 μg/mL showed a significant antioxidant activity, which was similar to that of Trolox^®^ (Figure 2). The Fenton reaction is an important source of hydroxyl radicals. This reaction is due to the combination of hydrogen peroxide and free iron inside the cell. In this study, we demonstrated that the RMHA extracts are able to quench hydroxyl radicals, and thinking about Fenton chemistry, one last question needed to be answered. Are the RMHA extracts capable of reducing and chelating iron thus minimizing the Fenton reaction? At all tested concentrations, RMHA proved to be efficient in reducing free iron, but only 10 and 100 µg/mL showed similar or better performance, respectively, to Trolox^®^ (Figure 3). The ferrous ion (Fe^2+^) is required for oxygen and electron transport and participates in the activities of many enzymes and the physiological concentrations in the human body are capable of participating in Fenton reactions, thus generating hydroxyl radicals [22]. The RMHA extracts were able to chelate Fe^2+^ at all tested concentrations, thus preventing the hydroxyl radical generation (Figure 4). It is important to mention that the aforementioned redox active metals are involved in neurodegenerative diseases, such as Alzheimer’s, where high iron concentrations were found in neural plaques, increasing the oxidative damage, whereas the amyloid beta deposition enhances the H_2_O_2_ generation and consequently the generation of hydroxyl radicals by Fenton chemistry. Based on these results, RMHA could represent a promising alternative to prevent or decrease the oxidative damage caused by the most reactive and harmful radical, the hydroxyl radical. 

NO**^·^** is an important mediator of acute and chronic inflammation by stimulation of cyclooxygenase (COX) activity resulting in unbalanced production of pro-inflammatory prostaglandins. The ability of RMHA to scavenge NO**^·^** was measured by quantifying the production of nitrite derived from sodium nitroprusside (SNP) by the Griess reaction. The data shows that 0.1 to 100 µg/mL concentrations of RMHA significantly decreased SNP-derived nitrite formation, indicating a potential role as a NO scavenging substance (Figure 5) and, therefore, it might regulate the production of these reactive species in biological systems. Rabelo *et al.* [15] have evaluated the antinociceptive and anti-inflammatory activity of aqueous extract of *Remirea maritima* and the results suggested that this extract has an antiinflammatory action probably mediated via inhibition of peripheral mediators (such as the synthesis of prostaglandins, NO, ROS). However, the aqueous extract only reduced NO formation at concentrations of 100 µg/mL to 1 mg/mL [15], while the RMHA showed significantly decreased nitric oxide production at 0.1 to 100 µg/mL and all of them were greater than Trolox^®^ (Figure 5). In neurodegenerative diseases the inflammatory scenario is considered a pivotal feature that contributes significantly to the increase in oxidative stress and hence the progression of the disease. A pro-inflammatory condition could lead to a nitric oxide overload, increasing the oxidative damage due to peroxynitrite formation.

Lipid peroxidation can be defined as a cascade of biochemical events resulting from the action of free radicals on the unsaturated lipids of cell membranes, which leads to alteration of permeability, loss of selectivity for input and/or output of nutrients and toxic substances to the cell, DNA changes, oxidation of LDL and commitment of extracellular matrix components [44]. RMHA at 1 to 100 µg/mL concentration was able to prevent the *in vitro* lipid peroxidation as evidenced from the TBARS assay (Figure 6A). Rabelo *et al.* [15] also tested the lipid peroxidation ability of aqueous extract of *Remirea maritima* and this extract significantly inhibited free radicals induced by TBARS at concentrations of 100 µg/mL to 1 mg/mL [15]. According to the medicinal plant literature, natural products which present a reduction in lipid peroxidation from 10 µg/mL are considered to have high antioxidant potential [36,37]. RMHA showed IC_50_ values of 7.5 µg/mL (Figure 6B), indicating a strong potential in decreasing lipid peroxidation *in vitro*.

To summarize, in this study, the RMHA extracts presented more efficient antioxidant activities in TRAP/TAR, FRAP, chelating activity, hydroxyl and nitric oxide scavenging activities compared with plant extracts or isolated compounds assessed by other groups, using the same approach used in the present work to investigate them [15,40,45,46,47,48].

Some studies consider that water/alcohol solvent is better than water solvent for the extraction of phenolic agents [6,49]. Since the antioxidant activity is related to total phenolic content, the RMHA redox properties are probably due to the presence of a high polyphenol content. 

Several polyphenol substances found in plant-derived diets have been shown to be able to inhibit proliferation and induce apoptosis in tumor cells [7,8,10]. One of the reasons for carcinogenesis is ROS production. In addition, ROS can specifically trigger certain pathways involved in tumor proliferation, contributing to genomic instability [26]. 

The global incidence of skin cancer around the world is high and 132,000 new melanoma skin cancers occur globally each year [50], thus, the RMHA was evaluated for cytotoxicity in normal cells and for the ability to inhibit the proliferation of melanoma cell lines. The selective index (SI) is often used to evaluate the differential toxicity of a compound, which is determined as a ratio between IC_50_ values of normal and cancerous cells. An SI value lower or equal of 2 suggests the general toxicity of pure compound in cells [51]. The SI for RMHA between fibroblast and melanoma cells was 0.65, therefore, the results showed that, although, it presented a significantly reduction of viable cells of B1610 melanoma cells at concentrations of 80 to 320 µg/mL, RMHA was not selective for melanoma skin cancer cells when compared with normal fibroblast cells (Figure 7).

It is important to mention that the IC_50_ value (70.96 µg/mL) of RMHA in reducing the fibroblast cell viability was 5.41, 29.56, 17.74 and 9.46 times higher than IC_50_ value shown in the *in vitro* antioxidants tests (EROs, IC_50_ 13.1 µg/mL; ferrous ion-chelating, IC_50_ 2.4 µg/mL; NO scavenging, IC_50_ 4.0 µg/mL and TBARS, IC_50_ 7.5 µg/mL, respectively), which does not preclude its applicability as an antioxidant agent since the therapeutic concentration is lower than the toxic concentration for normal cells.

A previous chemical characterization of RMHA extracts performed by our group identified the the compounds luteolin, vitexin and isovitexin. These compounds are all flavonoids, which were previously reported in the *Remirea* genus [15], and are known for their antioxidant properties [52,53,54].

Luteolin is a flavone found in a variety of plants, especially vegetables [55,56]. Previous studies showed stronger antioxidant properties and no change potential pro-oxidant activity of luteolin when compared to various common flavonoids, such as quercetin and myricetin, which suggests potential health benefits for humans [57].

Horváthová *et al.* [58] reported that luteolin has a protective effect against H_2_O_2_-induced DNA damage and possesses a protective effect on chromosomal aberrations induced by the cytostatic drug, melphalan, in metastasis of malignant melanoma cells. Besides, luteolin was able to induce cell apoptosis by modulating both the extrinsic pathway and intrinsic pathways, indicating that it triggers caspase-dependant apoptosis [59]. Previous studies have shown that luteolin also reduced the viability of diverse human cancer cell lines. In this study, this flavone was capable to increase the apoptotic of cells through caspase-3- and caspase-7-dependent pathways and was suggested that luteolin is a safe molecule with potential for clinical use in cancer therapy [60].

Isovitexin and vitexin are flavone glycosidea present in some drugs, medicinal plants and nutraceuticals [61]. Isovitexin is already known to exhibit antioxidant potential (inhibition of lipid peroxidation), to reduce the amount of hydrogen peroxide and to inhibit the production and, or release of tumor necrosis factor and prostaglandin E2 (PG2) in inflammatory processes induced by lipopolysaccharide (LPS) in mouse macrophages. These findings suggested that suppression of ROS-mediated COX-2 expression by isovitexin is beneficial in reducing inflammation and carcinogenesis [53]. 

Vitexin has already shown in different studies anti-oxidant [62], peripheral analgesic and central anti-inflammatory [63,64], anti-viral [65] and anti-convulsant [66] properties. In addition vitexin also showed anti-tumor and anti-metastatic activities and they were associated through a proapoptotic process, which is mediated by a decreased Bcl-2/Bax ratio and activation of caspases [67,68].

Based on this, it would be reasonable to suppose that the broad spectrum of *in vitro* antioxidant and anti-proliferative effects in melanoma cells presented by RMHA extracts in this study are due to these flavonoids alone or in association. Crude extracts generally consist of a mixture of several different compounds that can acting with antagonistic or synergistic effects. Therefore, it is interesting to suppose that the fractionation of the extract could eliminate toxic compounds which can become fractions with selective antiproliferative effects. 

## 4. Experimental Section

### 4.1. Chemicals

2,2′-Azobis(2-methylpropionamidine) dihydrochloride (AAPH), 5-amino-2,3-dihydro-1,4-phthalazinedione (luminol), 2-deoxy-D-ribose, glycine, Griess’ reagent, sodium nitroprusside (SNP), 2-thiobarbituric acid (TBA), 4,6-dihydroxypyrimidine-2-thiol, hydrogen peroxide (H_2_O_2_), dimethyl sulfoxide (DMSO), 2,4,6-tris(2-piridyl)-s-triazine, ferric chloride, and sodium acetate, were purchased from Sigma Chemical Co. (St. Louis, MO, USA). Materials used in cell culture were acquired from Gibco^®^/Invitrogen (São Paulo, SP, Brazil) and from the Rio de Janeiro Cell Bank (BCRJ, Rio de Janeiro, Brazil).

### 4.2. Plant Material and Preparation of R. maritima Extracts

*R. maritima* (the whole plant) was collected from the beach area of the city of Pirambu (Sergipe, Brazil; 10°55ʹS, 35°6ʹW), on February 2011 and it was identified by Prof. Ana Paula Prata, plant taxonomist, from the Department of Biology/UFS. A voucher specimen (No. ASE 20166) has been deposited in the Herbarium of Department of Biology, Federal University of Sergipe, São Cristóvão, Sergipe, Brazil. Permission for plant collection was obtained from the Chico Mendes Institute for Biodiversity Conservation of the Brazilian Ministry of the Environment (permit #25637-1). The whole plant was cleaned, dried and grounded into powder form. The hydroalcoholic extract of *R. maritima* (RMHA) were prepared by heating 15 g powder/300 mL EtOH/H_2_O (40% *v*/*v*) for 30 min followed by filtration, solvent extraction and lyophilization (RMHA yield: 6.2%). The extract redissolved readily in distilled water which was used as the vehicle. 

### 4.3. Determination of Total Phenolic Content (TPC)

The total phenolic content assay was performed using the Folin–Ciocalteu reagent, with the slight modification of Singleton *et al.* [69]. RMHA (1 mg) was diluted in water (1 mL). An aliquot (100 µL) of RMHA was then added to deionized water (6 mL) with the Folin–Ciocalteu reagent (500 µL). The mixture was shaken for 1 min. After addition of 15% Na_2_CO_3_ solution (2 mL), the mixture was shaken for 0.5 min. The solution was then diluted with deionized water to a final volume of 10 mL. After incubation for 120 min at 23 °C, the total phenolic content was determined at 750 nm using a spectrophotometer. Gallic acid was used as standard, and total phenolic content was expressed as (mg gallic acid equivalents/g of RMHA) estimated from a gallic acid calibration curve. The calibration curve range was 20–350 mg/mL (*R*^2^ = 0.999).

### 4.4. In Vitro Redox-Active Profile

#### 4.4.1. Total Reactive Antioxidant Potential (TRAP) and Total Antioxidant Reactivity (TAR) 

Total reactive antioxidant potential (TRAP) is an *in vitro* non-enzymatic method that is based on the action of antioxidants on the fluorescence decay of luminol-enhanced chemiluminescence generated by the reaction of luminol (o-aminophthaloylhydrazide) with the peroxyl radicals produced by thermal decomposition of the free radical generator AAPH [70,71]. First, the AAPH solution (120 mM final concentration) was prepared by adding the AAPH reagent in 100 mM glycine buffer pH 8.6 (20 mL final volume) followed by addition of luminol (4 µL, 0.001 mM final concentration) in the dark and then we allowed the system to stabilize for 2 h before the first reading [72]. Different concentrations of RMHA were added and the luminescence produced by the free radical reaction was quantified in a liquid scintillator counter (Wallac 1409, Perkin–Elmer, Boston, MA, USA) for 2 h. The system was considered the chemoluminescence emitted by AAPH thermolisys alone. The data were transformed in area under curve (AUC) calculated by software (GraphPad software^®^ San Diego, CA, USA; version 5.0) as previously described [73].

The Total Antioxidant Reactivity (TAR) readings were obtained in the same experiment. These results were calculated as the ratio of light intensity in the absence of samples (I_0_)/light intensity after RMHA addition [74].

#### 4.4.2. Hydroxyl Radical-Scavenging Activity 

This assay measures the ability of antioxidants to scavenge the hydroxyl radicals generated by the reaction between Fe^2+^ and H_2_O_2_ (Fenton reaction). The antioxidant capacity is indirectly determined by measuring (in the presence or absence of extracts) of the amount of malondialdehyde (MDA) generated by reaction between 2-deoxy-d-ribose and hydroxyl radical. The amount of MDA was determined by the absorbance of the chromophore generated in the reaction between MDA and thiobarbituric acid (TBA) measured at 532 nm [71,75].

The reactions were started by the addition of Fe^2+^ (FeSO_4_ 6 µM final concentration, 100 µL) to solutions containing 50 mM 2-deoxyribose (100 µL, 5 mM final concentration), 1 mM H_2_O_2_ (100 µL, 100 µM final concentration) and 20 mM of phosphate buffer (700 µL, pH 7.2).

To measure RMHA antioxidant activity against hydroxyl radicals, different concentrations (0.1 to 100 µg/µL) of RMHA were added to the system before Fe^2+^ addition. Reactions were allowed to proceed for 15 min at room temperature and then stopped by the addition of 4% phosphoric acid (*v*/*v*) followed by 1% TBA addition (*w*/*v*, in 50 mM NaOH, 500 µL). Solutions were boiled for 15 min at 95 °C, and then cooled to room temperature. After cooling, the absorbance was measured at 532 nm and results were expressed as percentage of MDA formed related to the reaction system.

#### 4.4.3. Ferric Reducing Antioxidant Power (FRAP)

This assay is used to determine the ability of iron reduction by antioxidants. Briefly, in a dark environment, we added each RMHA extract at the tested concentration (90 µL) to a screw tube followed by addition of distilled water (270 µL) and FRAP reagent (2.7 mL, 2.5 mL of 10 mM TPTZ, 2.5 mL of 20 mM Ferric Chloride and 25 mL of 0.3 M Acetate Buffer pH 3.6). After the mixture was homogenated and incubated in 37 °C for 30 min and readings were performed at 595 nm. A standard curve from a 5 mM ferrous sulphate (Fe^2+^ source) standard were performed to calculate the amount of Fe^2+^ produced during the reduction of Fe^3+^ by RMHA extracts.

#### 4.4.4. Fe^2+^ Chelation Assay 

The ferrous ion-chelating activity of RMHA was estimated as previously described by Cheng *et al.* [76]. Each RMHA concentration was incubated with 2 mM FeCl_2_ (50 μL) for 10 min. The reaction was initiated by adding 5 mM ferrozine (200 μL) followed by incubation for 5 min at room temperature. The ferrozine reacts with free iron yielding a red cromophore which absorbance is measured at 562 nm. EDTA (100 μg/mL), a standard chelating agent, served as the positive control. The Fe^2+^ chelating activity was calculated using the equation below:

Chelating activity (%) = (1 − Absorbance of sample/Absorbance of control) × 100
(1)

#### 4.4.5. Nitric Oxide (NO**^.^**) Scavenging Activity

Nitric oxide scavenging activity of RMHA extracts was determined from the decomposition of sodium nitroprusside in 20 mM phosphate buffer (pH 7.4) generating NO. Nitrite ions produced by interaction between the NO generated with oxygen were measured by the Griess reaction [77]. The reaction mixture is composed by 10 mM sodium nitroprusside (SNP) in phosphate buffer (pH 7.4) and RMHA at different concentrations totaling 1 mL final volume. This mixture were incubated at 37 °C for 1 h and after this, an aliquot of 0.5 mL was taken and homogenized with 0.5 mL Griess reagent. The absorbance of chromophore was measured at 540 nm. Results were expressed as percentage of nitrite formed compared to SNP alone representing 100% nitrite production. 

#### 4.4.6. Thiobarbituric Acid Reactive Species (TBARS) 

The thiobarbituric acid-reactive substances (TBARS) assay was employed to quantify lipid peroxidation [78]. The TBARS method is used to measure the antioxidant capacity of RMHA extract using egg yolk homogenate as lipid rich substrate [79]. First, egg yolk was homogenized (1% *w*/*v*) in 20 mM phosphate buffer (pH 7.4), sonicated at power 4 and 1 mL of emulsion was mixed with 0.1 mL of RMHA at different concentrations. Then, AAPH solution (0.1 mL, 0.12 M) was added as a peroxyl radical generating source to induce the lipid peroxidation. Incubation medium alone (egg yolk + PB 20 mM) without AAPH was used as control. Reactions were carried out at 37 °C. Samples were taken after 30 min and an aliquot (0.5 mL) were centrifuged with trichloroacetic acid (0.5 mL, final concentration 10%) at 10,000 *g* for 10 min. A portion of the supernatant (0.5 mL) was mixed with TBA (0.5 mL, 0.67%), heated at 95 °C for 30 min and cooled to room temperature. The sample’s absorbance was measured by using a spectrophotometer at 532 nm and the results were expressed as percentage of MDA formed compared to induced control (AAPH alone). 

### 4.5. Cells Line and Culture Conditions

The cytotoxicity assay of RMHA was performed using L929 (mouse fibroblasts) and B16F10 (melanoma) cell lines, all obtained from the Cell Bank of Rio de Janeiro, Brazil. Cells were grown in Dulbecco’s modified Eagle’s medium (DMEM), supplemented with 10% fetal bovine serum, 100 μg/mL streptomycin and 100 U/mL penicillin. The medium was replaced every two days and the cells were maintained at 37 °C in a 5% CO_2_ atmosphere. 

#### *In Vitro* Cytotoxicity Assay

Cytotoxic activity was performed by the neutral red assay. This method is based on the uptake of dye by the lysosomes of viable cells [80,81]. Cells were seeded at a density of 10^5^ cells/well into 96-well plates and incubated for 24 h at 37 °C and 5% CO_2_. Then, 20 µL of RMHA, at different concentrations (10–360 μg·mL^−1^) in water was added to the culture plates for 24 h. After treatment, cells were rinsed once with saline. Cells were then incubated for 3 h with neutral red solution (50 µg/mL in the well). Thereafter the medium was removed, the cells were washed quickly with an aqueous solution of 1% of formaldehyde and 1% of CaCl_2_, and then 200 µL of a solution of 1% acetic acid and 50% ethanol was added to each well to extract the dye. After agitation the plate was transferred to a microplate reader equipped with a 540 nm filter (Cary 50 Bio UV Visible, spectophotometer, Varian, Inc., Melbourne, Australia) to measure the absorbance. The cellular viability is express as the percentage of viable cells compared to the control group. For each assay, RMHA (6.25 mg·mL^−1^) was dissolved in distilled water and serial dilutions were obtained from this stock solution.

### 4.6. Statistical Analysis

The *in vitro* antioxidant assays were carried out with RMHA extract *n* = 3 (*i.e.*, 3 vials per group). The differences among data were evaluated by one-way analysis of variance (ANOVA) followed by Tukey’s post hoc test. The results were expressed as mean ± standard error of the mean (SEM) of three independent experiments. The cell culture experiments were performed with *n* = 4 and the results were expressed as mean ± standard error of the mean (SEM) of four independent experiments. The IC_50_ was calculated by non-linear regression fit analisys. In all cases differences were considered significant if *p* < 0.05. Data analyses were performed using the (GraphPad software^®^ San Diego, CA, USA; version 5.0).

## 5. Conclusions

In conclusion, the hydroalcoholic extract of *R. maritima* contains a high amount of total phenols and previous studies showed the presence of some flavonoids, which have potential anti-tumor, anti-metastatic, anti-inflammatory and anti-oxidant activities. Although these constituents are known to present several beneficial activities, until at the moment no study has demonstrated synergism of these compounds in their *in vitro* antioxidant and anti-proliferative activity on skin cells. The strong and efficient antioxidant properties of RMHA by different mechanisms are presented in this study for the first time. Some concentrations of this extract exhibited greater antioxidant capacity than the standard commercial antioxidant product Trolox^®^. Further, the promising results obtained in this study also suggest that *in vivo* approaches are needed to better evaluate the RMHA potential profile as a herbal medicine to prevent various diseases related to unbalanced production of oxygen and nitrogen reactive species. Additionally, it could serve as a chemopreventive agent or as adjuvant in chemotherapy against melanoma cells, however, further evaluations should be performed to confirm the anti- carcinogenic activity of this plant extract.
